# Cost-effectiveness of the anti-vascular endothelial growth factor intravitreal injection and panretinal photocoagulation for patients with proliferative diabetic retinopathy in South Korea

**DOI:** 10.1186/s12913-023-10280-6

**Published:** 2023-12-11

**Authors:** Hyeon-Jeong Lee, Songhee Cho, Jungeun Park, Yan Jin, Hyung Min Kim, Donghyun Jee

**Affiliations:** 1 Division of Healthcare Technology Assessment Research, National Evidence-based Healthcare Collaborating Agency, Seoul, South Korea; 2 Patient-Centered Clinical Research Coordinating Center, National Evidence-based Healthcare Collaborating Agency, Seoul, South Korea; 3 Department of Rehabilitation, College of Acupuncture and Moxibustion and Massage Health Preservation and Rehabilitation, Nanjing University of Chinese Medicine, Jiangsu, China; 4 Artificial intelligence, Kai Health, Seoul, South Korea; 5 Department of Ophthalmology and Visual Science, St. Vincent’s Hospital, Suwon, South Korea; 6 The College of Medicine, Catholic University of Korea, Seoul, South Korea

**Keywords:** Anti-VEGF injection, Cost-effectiveness analysis, Panretinal Photocoagulation, Proliferative diabetic retinopathy, South Korea

## Abstract

**Background:**

We determined the cost-effectiveness of the anti-vascular endothelial growth factor (VEGF) intravitreal injection versus panretinal photocoagulation (PRP) for patients with proliferative diabetic retinopathy (PDR) in South Korea.

**Methods:**

We simulated four treatment strategies using PRP and the anti-VEGF injection by constructing a Markov model for a hypothetical cohort of 50-year-old PDR patients: (1) PRP only; (2) anti-VEGF injection only; (3) PRP first; and (4) anti-VEGF injection first.

**Results:**

In this cost-effectiveness analysis, compared with only-PRP, the incremental cost-effectiveness ratio was $95,456 per quality-adjusted life-year (QALY) for PRP first, $34,375 per QALY for anti-VEGF injection first, and $33,405 per QALY for anti-VEGF injection only from a healthcare perspective. From the societal and payer perspective, strategy (2) was more cost-saving and effective than (1). In the probabilistic sensitivity analysis, only-PRP was cost-effective up to the willingness-to-pay (WTP) of about $42,000, while anti-VEGF injection only was cost-effective from a healthcare perspective. From the societal and payer perspectives, regardless of the value of WTP, anti-VEGF injection only was the most cost-effective strategy.

**Conclusion:**

In our study, the anti-VEGF injection for PDR was cost-effective from the payer and societal perspectives.

**Supplementary Information:**

The online version contains supplementary material available at 10.1186/s12913-023-10280-6.

## Background

Diabetic retinopathy (DR) is a leading cause of blindness worldwide [1]. Early detection and proper treatment can prevent vision loss from DR; thus, patients with diabetes should have regular follow-up with an ophthalmologist [2]. According to a systemic meta-analysis, the global prevalence of DR is about 28% in women and 25% in men [3]. With the prevalence of diabetes mellitus expected to rise from 463 million in 2019 to 700 million in 2045, the prevalence of DR is estimated to reach 160 million, and vision-threatening DR, in particular, 44 million in that period [4]. Among patients with DR, proliferative DR (PDR) carries a high risk of vision loss, even blindness. Hence, most treatment for DR is focused on treating or preventing PDR.

Panretinal photocoagulation (PRP) and the anti-vascular endothelial growth factor (VEGF) intravitreal injection are mainly used to prevent blindness due to DR [5]. Use of the anti-VEGF injection in DR has increased significantly [6–9]. However, both treatments have sharply contrasting pros and cons, which makes it difficult to make medical service choices [10–12]. While PRP has certain advantages—such as a fairly low cost, PDR treatment being covered by South Korea’s National Health Insurance Service (NHIS), and more long-term effects—PRP also has drawbacks, such as the visual field defect via peripheral retina atrophy after PRP [13, 14], and diabetic macular edema (DME), which may worsen visual acuity [15, 16]. The anti-VEGF injection does almost no harm to the retinal tissue, such as visual field damage and DME [7]. However, the economic burden of the anti-VEGF injection is heavy because the price for a single injection is expensive and not covered for PDR treatment in South Korea [17]. Moreover, the treatment effect is relatively short-term, the anti-VEGF injection must be repeated [18]. This inhibits the cost-effectiveness of the anti-VEGF injection, although this cost-effectiveness can vary according to the type of anti-VEGF agent involved [19].

As the pros and cons of the two approaches are clearly contrasting, the treatment is chosen according to the patient’s preference or the doctor’s choice in actual clinical practice. As such, it is necessary to provide evidence for medical service decisions regarding the economic evaluation of PRP and the anti-VEGF injection. We assess the treatment of PDR on an economic basis, including PRP and the anti-VEGF injection, using cost-effectiveness analysis.

## Method

Table 1 outlines our analysis, and Table 2 presents the values and references of all parameters input to the model.

**Table 1:** The outline of economic evaluation

Items	Contents
Population	50-year-old patients with proliferative diabetic retinopathy
Treatment strategy	∙ PRP only (covered by NHI in South Korea) ∙ PRP first ∙ Anti-VEGF injection only ∙ Anti-VEGF injection first
Decision analysis model	∙ Markov model
Time horizon	∙ 50 ~ 100 years old
Analysis cycle length	∙ 1 year
Perspectives	∙ Healthcare system perspective ∙ Payer perspective ∙ Societal perspective
Cost	Medical cost, non-medical cost (transportation, care cost), productivity loss cost (patient time cost)
Outcome	Quality-adjusted life year gained

PRP, panretinal photocoagulation; NHI, national health insurance; anti-VEGF, anti–vascular endothelial growth factor

**Table 2:** Model parameters: baseline values, ranges, and distributions for sensitivity analysis

Markov state transition probability	Baseline value	Range	Reference	Distribution
From NPDR to DME	0.152	20% - to 20% +	Hospital data	Beta
From PDR to DME	0.179	20% - to 20% +	Hospital data	Beta
From PDR to SVL	0.18	20% - to 20% +	Tung et al. (2006) [20]	Triangle
**Probability**				
Success rate of PRP for PDR	0.2008	20% - to 20% +	Hospital data	Beta
PDR Recurrence after PRP	0.0169	20% - to 20% +	Hospital data	Beta
Success rate of anti-VEGF injection to DME	0.534	20% - to 20% +	Wells et al (2015) [21]	Beta
Incidence of endophthalmitis after anti-VEGF injection	0.0006	20% - to 20% +	Fileta et al. (2014) [22]	Beta
Success rate of endophthalmitis treatment	0.595	20% - to 20% +	Fileta et al. (2014) [22]	Beta
Loss of follow-up for PDR treatment	0.2	20% - to 20% +	Assumption	Triangle
Follow-up continuation in SVL state	1st year 0.914 2nd year 0.736 3rd year 0.65 4th year 0.609 5th year 0.537 after 5th year 0.481	20% - to 20% +	NHIS claims data	NA
Annual discount rate	4.5%	3%, 7%	Bae et al. (2022) [23]	NA
**Relative risk**				
Effectiveness of anti-VEGF injection to PDR vs. PRP	1.75	95% CI 1.12 to 2.75	Gao et al. (2020) [24]	Lognormal
PDR Recurrence after anti-VEGF injection vs. PRP	1.15	95% CI 0.63 to 2.12	Sivaprasad et al. (2017) [25]	Lognormal
Death in diabetes patients vs. general population	1.49	95% CI 1.45 to 1.54	Shin et al. (2018) [26]	Lognormal
Death in SVL states vs. non-SVL state	1	1.54	Choi et al. (2020) [27]	Lognormal
**Cost**				
NPDR state per year		20% - to 20% +	NHIS claims data	Gamma
Medical cost (covered/non-covered)	160/12			
Transportation cost	4			
Time cost	82			
PDR state per year		20% - to 20% +	NHIS claims data	Gamma
Medical cost	348/44			
(covered/non-covered)				
Transportation cost	10			
Time cost	147			
DME state per year		20% - to 20% +	NHIS claims data	Gamma
Medical cost (covered/non-covered)	1,732/267			
Transportation cost	24			
Time cost	375			
SVL state per year		20% - to 20% +	NHIS claims data	Gamma
Medical cost (covered/non-covered)	338/49			
Transportation cost	12			
Time cost	162			
Care cost	11,826			
Low vision glasses	27			
PRP per episode		20% - to 20% +	NHIS claims data	Gamma
Medical cost (covered/non-covered)	188/24			
Transportation cost	2			
Time cost	32			
Anti-VEGF injection per episode		20% - to 20% +	NHIS claims data	Gamma
Medical cost (covered/non-covered)	447/63			
Transportation cost	2			
Time cost	25			
Endophthalmitis treatment per episode		20% - to 20% +	NHIS claims data	Gamma
Medical cost (covered/non-covered)	2,143/1,208			
Transportation cost	2			
Time cost	232			
**Utility**				
NPDR PDR DME SVL	0.849–0.904 0.846–0.901 0.857–0.912 0.796–0.851 (Adjusted to age)	20% - to 20% + (If exceed 1, censored to 1)	Survey	Triangle
Disutility by visual field defect after PRP	-0.01	-0.02 to -0.005	Expert opinion	NA

anti-VEGF, anti-vascular endothelial growth factor; CI, confidence interval; DME, diabetic macular edema; NHIS, National Health Insurance Service; NPDR, non-proliferative diabetic retinopathy; PDR, proliferative diabetic retinopathy; PRP, panretinal photocoagulation; RR, relative risk; SVL, severe visual loss

### Target population

The target population consisted of 50-year-old patients with PDR owing to the rising prevalence of DR in adults aged ≥ 50 years.

### Treatment strategies

We developed four strategies for the economic evaluation: (1) *PRP only*: using only PRP, covered by South Korea’s National Health Insurance Service (NHIS); (2) *anti-VEGF injection only*: using the anti-VEGF injection only; (3) *PRP first*: using PRP first, and if the treatment does not work, then using the anti-VEGF injection; and (4) *anti-VEGF injection first*: using the anti-VEGF injection first, and if the treatment does not work, then using PRP. In the management of PDR, patients may undergo treatment modifications if they show inadequate response to the initial intervention. For example, if the initial anti-VEGF injection fails to effectively arrest the progression of PDR, PRP may be employed. Furthermore, in cases where macular edema occurs despite initiating PRP, the addition of anti-VEGF injection might be considered. The response rate for the initial intervention was based on data extracted from anonymized records across six hospitals in South Korea and relevant previous studies (details described in ‘Model parameters’ section). Although more than four treatment patterns are used in actual clinical practice, it was not feasible to model them as they are, and we set four strategies as the treatment approach. We compared the PRP-only strategy to the other three other strategies. In clinical practice, it is common to alternately use PRP and the anti-VEGF injection according to the patient’s condition (rather than using either) for PDR. However, since only PRP is covered under the NHIS for PDR in South Korea, we used this strategy to compare with other strategies. Among all of them, we employed the three anti-VEGF injections as the loading dose.

### Model structure

**Fig. 1:**
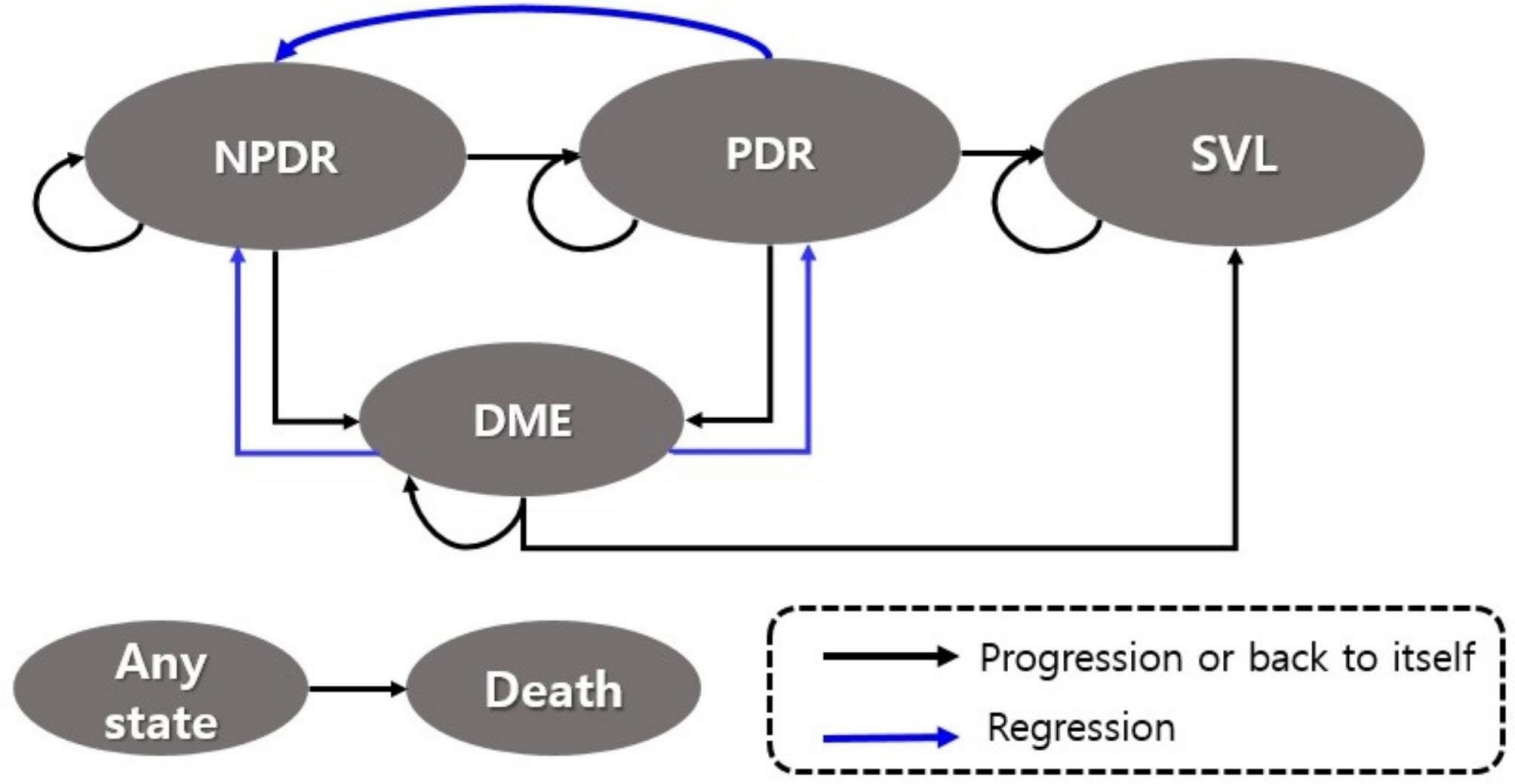
Schematic diagram for the transition of health states of diabetic retinopathy in the Markov model. Ovals indicate the Markov states; arrows denote transitions. NPDR, non-proliferative diabetic retinopathy; PDR, proliferative diabetic retinopathy; DME, diabetic macular edema; SVL, severe visual loss

For the economic evaluation, we built a Markov model and performed cohort simulation (Fig. 1). The Markov states consisted of non-proliferative DR (NPDR), PDR, DME, severe visual loss (SVL), and death based on the natural history of the disease. In the NPDR state, regular follow-up is performed. In the PDR state, PRP or the anti-VEGF injection is used. In the DME state, the anti-VEGF injection is used. SVL was defined as less than 0.1 visual acuity.

Response to treatment may be different in either eye, but if this is reflected in the model, the model may become overly complicated. We used the monocular model, which creates the model based on the eye with the better eyesight, as in prior studies [28]. As a major side effect of treatment for PDR, we modeled the DME and permanent peripheral visual field loss after PRP, and endophthalmitis after the anti-VEGF injection.

### Model assumptions

We assumed that the anti-VEGF injection would be given twice a year in the SVL state to avoid exacerbating the condition, leading to non-light perception. We calculated the treatment continuity rate in the SVL state from the NHIS claims data and applied it to the model. The NHIS is a compulsory government-run medical insurance system in South Korea, providing coverage to nearly the entire South Korean population. Approximately 97.2% of South Koreans are enrolled in the NHIS, and as a result, the NHIS database effectively represents the entirety of the Korean population [29]. We defined treatment continuation in the SVL as a case of outpatient treatment at least twice a year. We assumed that patients in the SVL state would receive care for three hours a day for daily living assistance. In this model, we considered care costs in cases of SVL state. Approximately 20% of men and 30% of women aged 65 and over require assistance with the activity of daily living [30]. So in the case of blindness, the care cost until death may be overestimated. We assumed that care was needed from the age of 90 even if blindness did not occur, and in order to avoid overestimating the cost, the care cost was calculated up to the age of 90 in the case of blindness.

### Cohort simulation

Our model involves a 100,000 hypothetical cohort of PDR patients aged 50 years. In the cohort simulation, patients start with the PDR state. If the treatment is successful and the angiogenesis is fully annihilated in PDR, they move on to the NPDR state. If neovascularization recurs, they move on to the PDR state. The patients in the PDR and NPDR states can shift to the DME state. Patients in any state can remain in that state or move to the death state. The Markov state changes via transition probabilities between the states (Fig. 1). Cohort simulation is terminated when the patients reach 100 years of age.

### Model parameters

#### Treatment-related probability and mortality

We calculated the treatment-related probability of PRP and the anti-VEGF injection using anonymized data from the Clinical Data Warehouse (CDW) of South Korea’s Catholic Medical Center. The Catholic Medical Center began operating the CDW to harness clinical data from six affiliated hospitals in 2019. We utilized data relating to patients diagnosed with DR from January 1, 2012, to April 1, 2019. In addressing incomputable probabilities with the CDW data, we utilized two approaches: reviewing relevant literature, including previous studies providing valuable insights, and analyzing NHIS claims data. The success rate of PRP for PDR and the PDR recurrence rate after PRP were calculated from CDW data. Additionally, the success rate of anti-VEGF injection for DME [21], the incidence of endophthalmitis after anti-VEGF injection [22], the success rate of endophthalmitis treatments [22], effectiveness of anti-VEGF injection to PDR vs. PRP [24] and PDR recurrence after anti-VEGF injection vs. PRP [25] were extracted from a randomized controlled study (RCT), systematic reviews, and a meta-analysis. Since the target population was DR patients with diabetes, we applied the risk ratio (RR) of death from diabetes to the mortality rate of the general population, which was calculated from the National Health Insurance Service-Health Screening Database of South Korea [26].

### Cost

We opted for a real-world data-based approach to calculate costs. Using the NHIS claims data, we calculated the annual cost for each Markov state and the treatment cost of the PRP and the anti-VEGF injection per episode and the cost was calculated using the macro-costing method, which determines the cost per episode rather than using micro-costing based on individual treatment costs and frequency. We identified cost items according to various perspectives. From the payer perspective, only medical expenses covered by the NHIS were included. From the healthcare system perspective, medical costs incurred within the healthcare system were included (i.e., medical expenses covered and not covered by the NHIS). From the societal perspective, medical, transportation, time, and care costs were included. We computed covered medical expenses by analyzing claims data from the NHIS; estimated non-covered medical costs by calculating the non-coverage rate from South Korea’s Health Insurance Patient Medical Expenses Survey; determined transportation costs based on 2018 data from the annual South Korea Medical Panel Survey (2008–2018) (Version 1.7); established the time costs using the hourly wage for outpatients and the daily wage for hospitalized patients from South Korea’s Ministry of Employment and Labor; and used the employment rate from the country’s Economic Activity Population Survey. Costs are expressed in 2020 US dollars (USD). If no data for 2020 were available, we employed the data for the nearest year, an relied on the consumer price index to adjust the costs for 2020.

### Utility

We assessed utility using the EuroQol five-dimensions with a 3-level questionnaire (EQ-5D-3 L), a generic health-related quality of life (HRQoL) measurement tool [31]. Data on HRQoL for DR patients are scarce in South Korea. We performed a survey to evaluate the HRQoL for 300 DR patients at St. Vincent’s Hospital, run by the Catholic University of South Korea. Based on previous studies focusing on visual field defects and the decline in the utility among glaucoma patients, it has been substantiated that visual field impairment leads to a reduction in their quality of life [32, 33]. However, we could not locate studies specifically addressing the extent of utility decrease resulting from peripheral visual field defects. Because no previous study had been conducted on the HRQoL of PDR patients with peripheral visual field defect (pVFD) in South Korea, we assumed that utility would decrease due to permanent pVFD, which we set to 0.01 based on expert opinion.

### Analysis

We computed the incremental cost-effectiveness ratio (ICER) as a final outcome of the model using quality-adjusted life years (QALYs) to represent effectiveness. Given the uncertainty of the parameters, we performed deterministic and probabilistic sensitivity analyses (Range of sensitivity analyses shown in Table 2). For deterministic sensitivity analysis, we carried out one-way sensitivity analysis for each parameter in the model; the results are presented in tornado diagrams, which comprise an integrated set of one-way sensitivity analyses. Probabilistic sensitivity analysis caused the parameters to vary simultaneously based on probability distributions with 10,000 re-samplings. The results are presented in a cost-effectiveness acceptability curve (CEAC). We conducted our analyses using TreeAge Pro Healthcare 2020 (TreeAge software, MA, US).

## Results

### Base case results

From the healthcare system perspective, the expected cost of the PRP-only strategy for one patient was $9,153; for the PRP-first strategy, it was $10,894; for the anti-VEGF injection-first strategy, it was $12,834; and for the anti-VEGF injection-only strategy, it was $15,446. Compared with the PRP-only strategy, there was an additional cost of $1,741 for the PRP-first strategy; $3,681 for the anti-VEGF injection-first strategy; and $6,293 for the anti-VEGF injection-only strategy. The effectiveness was 13.07 QALYs for the PRP only strategy, 13.09 QALYs for the PRP first strategy, 13.18 QALYs for the anti-VEGF injection first strategy, and 13.26 QALYs for the anti-VEGF injection only strategy. Compared with the PRP only strategy, a patient who received the PRP first strategy gained 0.02 more QALYs, a patient who received the anti-VEGF injection first strategy gained 0.11 more QALYs, and a patient who received the anti-VEGF injection only strategy gained 0.19 more QALYs. The ICER of the PRP first strategy over the PRP only strategy was $95,456 per QALY; for the anti-VEGF injection first strategy, it was $34,375 per QALY; and for the anti-VEGF injection only strategy, it was $33,405 per QALY. Hence, during a lifetime horizon, the anti-VEGF injection only strategy was dominant. As the threshold ICER of $24,400 per QALY [34]—which has been suggested as willingness-to-pay (WTP) per one QALY gain in South Korea—the anti-VEGF injection only strategy was not cost-effective.

From the societal and payer perspectives, the anti-VEGF injection only, the anti-VEGF injection first, and the PRP-first strategies were dominant methods to save costs and increase effectiveness compared with the PRP-only strategy. The anti-VEGF injection-only strategy was the least costly and the most effective compared with the other strategies (Table 3).

**Table 3:** Cost-effectiveness results from base case

Strategy	Cost ($)	Incremental Cost ($)	Effectiveness (QALY)	Incremental Effectiveness (QALY)	Incremental cost-effectiveness ratio ($/QALY)
**Healthcare system perspective**					
PRP only	9,153		13.07		
PRP first	10,894	1,741	13.09	0.02	95,456
Anti-VEGF first	12,834	3,681	13.18	0.11	34,375
Anti-VEGF only	15,446	6,293	13.26	0.19	33,405
**Societal perspective**					
PRP only	85,188		13.07		
PRP first	81,536	-3,653	13.09	0.02	Cost-saving
Anti-VEGF first	76,737	-8,451	13.18	0.11	Cost-saving
Anti-VEGF only	70,366	-14,823	13.26	0.19	Cost-saving
**Payer perspective**					
PRP only	5,132		13.07		
PRP first	4,477	-656	13.09	0.02	Cost-saving
Anti-VEGF first	4,053	-1,079	13.18	0.11	Cost-saving
Anti-VEGF only	3,361	-1,772	13.26	0.19	Cost-saving

QALY: quality-adjusted life year; PRP: panretinal photocoagulation; anti-VEGF: anti-vascular endothelial growth factor

### Sensitivity analysis

**Fig. 2:**
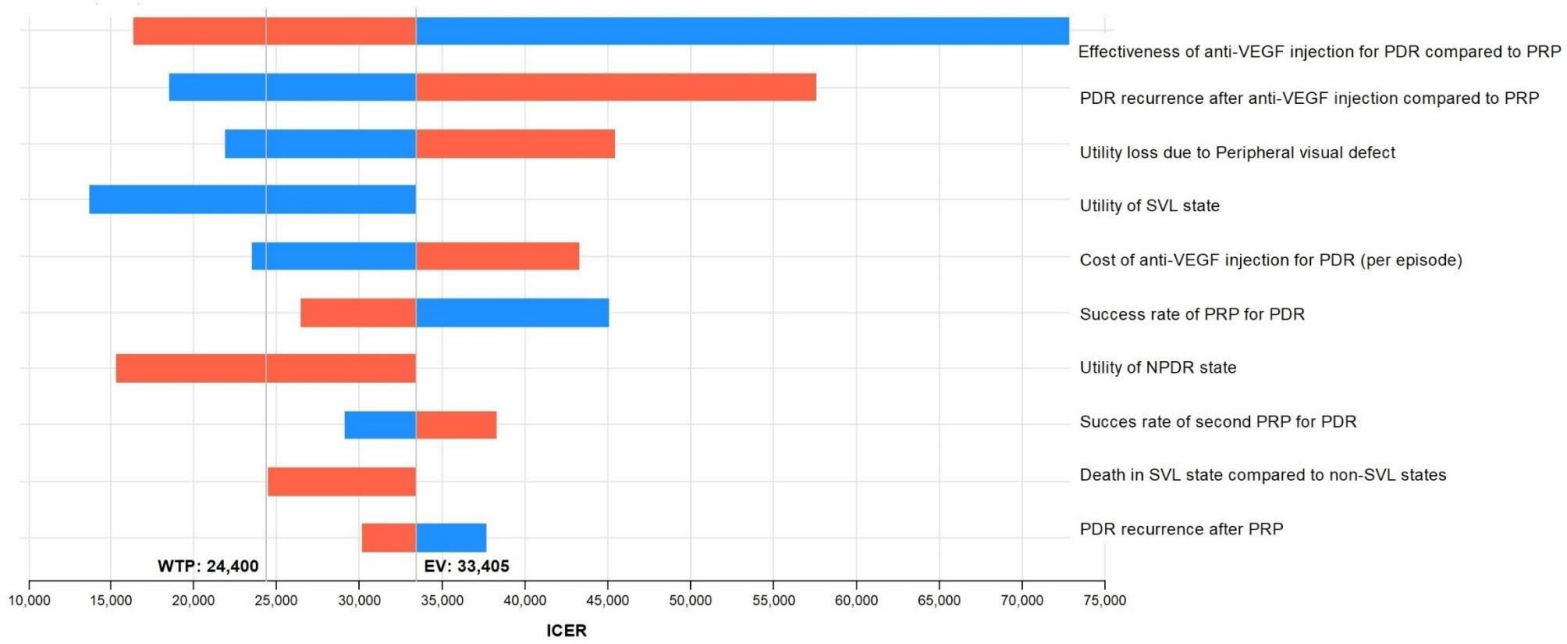
Tornado diagram of anti-VEGF injection only strategy versus PRP only strategy from the healthcare perspective. Each horizontal bar is generated from each parameter being analyzed. The blue portion indicates the ICER change when the parameter value falls below its base value, and the red portion refers to the ICER change when the parameter rises above its base value

In the sensitivity analysis, we compared the anti-VEGF injection-only strategy versus the PRP-only strategy from the healthcare perspective. We conducted one-way sensitivity analysis for the anti-VEGF injection-only strategy, which had the smallest ICER compared with the PRP-only strategy. The ICER of the anti-VEGF injection-only strategy over the PRP-only strategy fell below the threshold ICER of $24,400 per QALY when altering the following parameters: increased effectiveness of the anti-VEGF injection compared with PRP; decreased recurrence of PDR after receiving the anti-VEGF injection compared with PRP; decreased utility loss due to pVFD after PRP; decreased SVL state utility; decreased cost of the anti-VEGF injection for PDR per episode; and increased NPDR state utility. The tornado diagram between the PRP only strategy and the anti-VEGF injection only strategy is shown in Fig. 2.

**Fig. 3:**
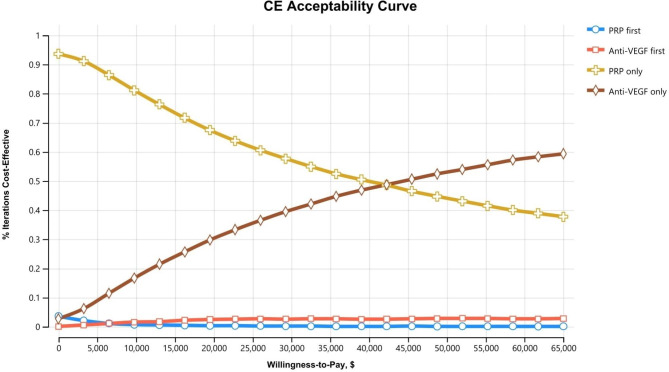
The cost-effectiveness acceptability curve from the probabilistic sensitivity analysis based on the healthcare perspective. It denotes the probability of being cost-effective (the most effective option within a threshold ICER) at a particular WTP value. This cost-effectiveness acceptability curve was plotted from the probabilistic sensitivity analysis of 10,000 trials in four strategies

**Fig. 4:**
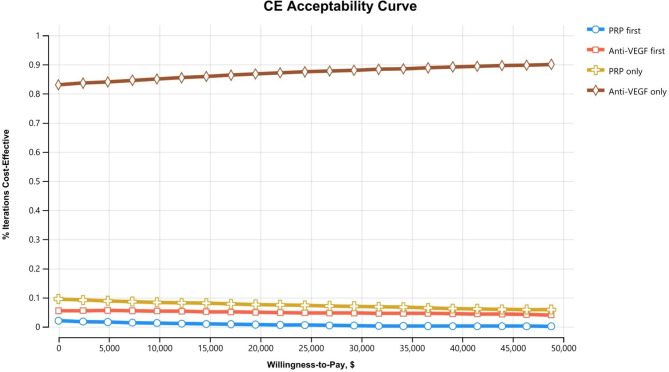
The cost-effectiveness acceptability curve from the probabilistic sensitivity analysis based on the societal perspective. It denotes the probability of being cost-effective (the most effective option within a threshold ICER) at a particular WTP value. This cost-effectiveness acceptability curve was plotted from the probabilistic sensitivity analysis of 10,000 trials in four strategies

**Fig. 5:**
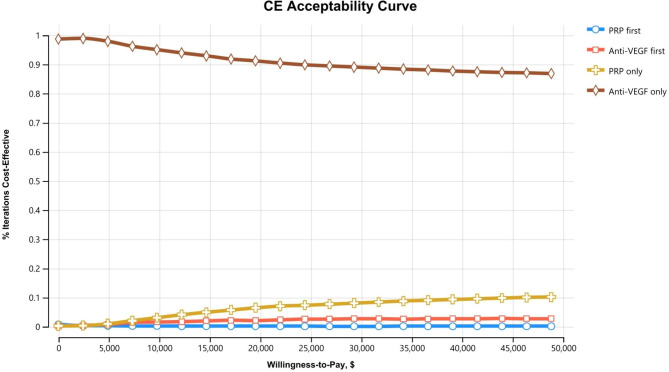
The cost-effectiveness acceptability curve from the probabilistic sensitivity analysis based on the payer perspective. It denotes the probability of being cost-effective (the most effective option within a threshold ICER) at a particular WTP value. This cost-effectiveness acceptability curve was plotted from the probabilistic sensitivity analysis of 10,000 trials in four strategies

In the probabilistic sensitivity analysis from the healthcare perspective, the PRP-only strategy was most likely to be cost-effective in the range of up to about $42,000 in WTP, and the anti-VEGF injection-only strategy was most likely to be cost-effective in the WTP thereafter (Fig. 3). In the probabilistic sensitivity analysis from the societal and payer perspectives, regardless of the value of WTP, the anti-VEGF injection-only strategy was the most cost-effective strategy (Figs. 4 and 5).

### Discussion and conclusion

We performed an economic evaluation of the anti-VEGF injection and PRP for treatment of PDR through cost-effectiveness analysis using Markov modeling. From the healthcare system perspective, compared with the PRP-only strategy, the ICER of the anti-VEGF injection-only strategy was $33,405 per QALY. The ICER of the anti-VEGF-first strategy was $34,375 per QALY compared with the PRP-only strategy. In the probabilistic sensitivity analysis, the PRP-only strategy was cost-effective up to the WTP of about $42,000, while the anti-VEGF injection-only strategy was cost-effective thereafter. From the societal and payer perspectives, the anti-VEGF injection-only, the anti-VEGF injection-first, and the PRP first strategies (compared with the PRP-only strategy) were cost-saving methods, while the anti-VEGF injection-only strategy was the least costly and the most effective approach compared with the other strategies. In the probabilistic sensitivity analysis, regardless of the value of WTP, the anti-VEGF injection only strategy was the most cost-effective technique.

Anti-VEGF injection treatment showed a cost-saving effect from the societal and payer perspectives owing to the following reasons. The medical cost for PDR was more than twice the cost of NPDR ($348 for PDR versus $160 for NPDR). When DME occurred, the medical cost exceeded $1,700. Most patients in the anti-VEGF treatment group remained in the NPDR state, whereas many patients in the PRP treatment group progressed to the SVL state. This indicates that anti-VEGF treatment reduces the severity of DR, suppresses progression to the SVL state, and has the advantage of preventing DME. Hence, although anti-VEGF treatment is more expensive, the cost-saving effect of reducing the severity of DR and preventing DME in PDR treatment is much greater. From the societal perspective, cost reduction effects (including time, transportation, and care costs) are also added. Anti-VEGF treatment is not cost-effective from the healthcare system perspective owing to the high cost of non-covered anti-VEGF injection.

In a previous study, Hutton et al. performed a cost-effectiveness analysis for PDR treatment between PRP and the ranibizumab injection based on the clinical trial of Protocol S by DRCR.net [28]. The ICER of the ranibizumab injection to PRP for PDR with DME was $55,568 per QALY, whereas the ICER for PDR without DME was $662,978 per QALY. The difference between our study and Hutton’s is that Hutton built a PDR treatment model that depends on the presence or absence of DME, but we used Markov modeling in which we applied the occurrence of DME for every year. Another difference is that the ICER in our study is lower than the one in Hutton’s. One reason could be that our low ICER may be due to the lower medical costs of South Korea than those found in the US [35–38]. Hutton indicated that if the price of ranibizumab (which has the greatest effect on ICER) fell from $1,916 to $900, ranibizumab would become more cost-saving than the combined treatment of PRP and ranibizumab in patients with DME. However, the cost of anti-VEGF treatment in South Korea does not exceed $900; in this backdrop, our conclusion that the anti-VEGF strategy is cost-saving may be reasonable.

In actual clinical practice, a combination of PRP and the anti-VEGF injection is frequently used to treat PDR, and the use of either PRP or the anti-VEGF injection is rare [39]. If a frequent change in treatment between PRP and the anti-VEGF injection were applied to the Markov modeling, the model would become overly complicated, creating challenged in interpreting the results. When interpreting the results of the economic evaluation, it is difficult to conclude that any one of the four strategies is dominant and cost-effective. Thus, we condensed the numerous treatment options into four treatment strategies.

Many studies have attempted to measure HRQoL using the EQ-5D in patients with eye diseases, but the EQ-5D may be less sensitive for this subset of the population [40–42]. In our study, the HRQoL of patients with blindness was not significantly different from that of other health states in the Markov model. This may be due to the EQ-5D’s lack of sensitivity to eye disease, or it may be due to the patient’s adaptation to blindness. A recent study, which reported the EQ-5D’s utility using data from the South Korea National Health and Nutrition Examination Survey (2008–2012), found that the utility of severe visual impairment was 0.894 [43]. We also assessed the HRQoL of DR patients based on the National Eye Institute Visual Function Questionnaire, which contains 25 items (NEI-VFQ-25) [44]. We found it to be significantly lower in the blindness state than in other health states (data not shown here). Since the EQ-5D does not sensitively reflect the HRQoL of DR patients, the ICER of the anti-VEGF injection (compared with PRP) might be overestimated.

The effectiveness of anti-VEGF therapy may be underestimated in our study because the EQ-5D has no vision-related questions. The HRQoL of the blindness state had a great influence on our outcomes. We performed additional analysis that involved the Health Utility Index 3 tool, including a visual acuity question from previous literature [40] regarding HRQoL and found that the ICER of the anti-VEGF injection-only strategy (compared with the PRP-only method) fell from $33,405 per QALY to $9,209 per QALY.

Our study has several limitations. The Markov model assumes that DR in both eyes will progress with the same severity and at the same response rate to the treatment. Since diabetes is a systemic illness, it is reasonable to conclude that diabetes affects the retina in both eyes, but the severity of DR can be different between the eyes in a clinical setting. Another limitation is that some parameters from the anonymized hospital data showed differences from the values ​​reported in prior research. This may be because the data were sourced from only a few medical institutions (not all patients) in South Korea, or to the low validity of hospital-based data by the loss of follow-up and transfer to other hospitals. To compensate for this limitation of hospital-based data, for most parameters where South Korea data were not available, we utilized data from randomized controlled trials (RCTs) or meta-analysis studies of RCTs to enhance the robustness of the input parameters. Among these parameters, the effectiveness of anti-VEGF injection versus PRP for PDR and PDR recurrence after anti-VEGF injection versus PRP were found to significantly impact the ICER based on one-way sensitivity analysis. This sensitivity analysis highlights the substantial uncertainty associated with the study findings, emphasizing the need for cautious interpretation and the importance of further research to validate and refine the model’s parameters.

Our study has limitations in the disutility of pVFD. Although our one-way sensitivity analysis demonstrated that the utility decrement associated with pVFD significantly influenced ICER results, it is important to acknowledge that this parameter was based on expert opinion due to the limited availability of empirical data. Consequently, this reliance on expert opinion may introduce potential uncertainties, affecting the overall robustness of our findings. Further research to obtain empirical evidence on the pVFD’s impact on utility would inform cost-effective treatment decisions for PDR patients.

In sum, although anti-VEGF injection is frequently used as an effective alternative to PRP treatment, the anti-VEGF injection has several limitations, such as a high cost and repeated injections. In our study, the anti-VEGF injection for PDR was cost-effective from the payer and societal perspectives. Our results on the cost-effectiveness of the anti-VEGF injection for PDR, alone or in combination with PRP treatment, can be used as important evidence when making medical service decisions.

### Electronic supplementary material

Below is the link to the electronic supplementary material.

Supplementary Material 1

## Data Availability

The datasets used during the current study are available from the corresponding author on reasonable request.

## Abbreviations

anti-VEGF: anti-vascular endothelial growth factor
CDW: Clinical Data Warehouse
CEAC: Cost-effectiveness acceptability curve
DME: Diabetic macular edema
DR: Diabetic retinopathy
EQ-5D-3L: EuroQol five-dimensions with a 3-level questionnaire
HRQoL: Health-related quality of life
ICER: Incremental cost-effectiveness ratio
NEI-VFQ-25: National Eye Institute Visual Function Questionnaire with 25 items
NHIS: National Health Insurance Service
NPDR: Non-proliferative DR
PDR: Proliferative DR
PRP: Panretinal photocoagulation
pVFD: Peripheral visual field defect
QALY: Quality-adjusted life year
RR: Risk ratio
SVL: Severe visual loss
USD: US dollar
